# CASE SERIES OF PATIENTS UNDER BIWEEKLY TREATMENT WITH LARONIDASE: A REPORT OF A SINGLE CENTER EXPERIENCE

**DOI:** 10.1590/1984-0462/;2019;37;3;00010

**Published:** 2019-05-09

**Authors:** Sandra Obikawa Kyosen, Leny Toma, Helena Bonciani Nader, Marion Coting Braga, Vanessa Gonçalves Pereira, Sueli Canossa, João Bosco Pesquero, Vânia D’Almeida, Ana Maria Martins

**Affiliations:** a Universidade Federal de São Paulo, São Paulo, SP, Brazil.

**Keywords:** Enzyme replacement therapy, Metabolism, inborn errors, Glycosaminoglycans, Mucopolysaccharidosis I, Terapia de reposição de enzimas, Erros inatos do metabolismo, Glicosaminoglicanos, Mucopolissacaridose I

## Abstract

**Objective:**

To report the stabilization of urinary glycosaminoglicans (GAG) excretion and clinical improvements in patients with mucopolysaccharidosis type I (MPS I) under an alternative dose regimen of laronidase of 1.2 mg/kg every other week.

**Methods:**

We participated in a dose-optimization trial for laronidase in MPS-I patients using four alternative regimens: 0.58 mg/kg every week, 1.2 mg/kg every two weeks, 1.2 mg/kg every week and 1.8 mg/kg every other week (EOW). After the trial ended, the patients resumed the recommended dose and regimen of 0.58 mg/kg every week. Under this regimen, some patients presented difficulties in venous access and were unable to commute weekly to the treatment center. Therefore, we used an alternative regimen that consisted of 1.2 mg/kg EOW in eight patients. A retrospective study of medical records of MPS-I patients who underwent both enzyme replacement therapy (ERT) regimens, of 0.58 mg/kg every week and 1.2 mg/kg EOW, was done.

**Results:**

Patients remained clinically stable under the alternative regimen, did not present elevation of urinary GAG nor any adverse event.

Conclusions: The switch of dose regimen to 1.2 mg/kg EOW of laronidase was safe, and did not cause any clinical worsening in patients who had been previously under standard dose ERT.

## RESUMO

**Objetivo:**

Descrever a manutenção dos níveis de glicosaminoglicano (GAG) excretados na urina e da estabilização clínica em pacientes com mucopolissacaridose do tipo I (MPS I) com o uso da laronidase num regime de dose alternativo de 1,2 mg/kg a cada duas semanas.

**Método:**

Alguns pacientes do nosso serviço participaram de um estudo de otimização de dose da laronidase para o tratamento da MPS I no qual foram comparados quatro esquemas terapêuticos: 0,58 mg/kg/semana, 1,2 mg/kg a cada duas semanas, 1,2 mg/kg/semana e 1,8 mg/kg a cada duas semanas. Após o término do estudo, todos os pacientes passaram a receber a terapia de reposição enzimática (TRE) na dose padrão de bula, que é de 0,58 mg/kg/semana, e nesse regime alguns pais se queixaram da dificuldade em comparecer ao centro todas as semanas, além da dificuldade de se obter acesso para punção venosa. Com base nessas queixas, oito pacientes passaram a receber a TRE no regime alternativo de 1,2 mg/kg a cada duas semanas. Foi feito o estudo retrospectivo de dados de prontuário de pacientes com MPS I que fizeram TRE com laronidase nas doses 0,58 mg/kg/semana e 1,2 mg/kg a cada duas semanas.

**Resultados:**

Os pacientes mantiveram-se clinicamente estáveis, não apresentaram aumento dos níveis de GAG urinários nem eventos adversos durante o regime alternativo de dose.

**Conclusões:**

A mudança para o esquema de 1,2 mg/kg de laronidase a cada duas semanas foi segura e não acarretou piora clínica nos pacientes que já estavam em TRE na dose padrão.

## INTRODUCTION

Mucopolysaccharidosis type I (MPS I) is a genetic disease from the lysosomal storage group, with an autosomal recessive inheritance caused by mutations in the *IDUA* gene, which reduce the activity of the enzyme α-L-iduronidase.^1^ This enzyme deficiency leads to an accumulation of glycosaminoglycans (GAGs) - particularly heparan sulfate and dermatan sulfate -, which results in varying degrees of damage to cells, tissues, and organs.^1^ MPS I has an estimated incidence of one case per 100 thousand live births^1^ ^,^ ^2^ ^,^ ^3^ and wide clinical variability, being classified according to their phenotypic presentation into severe form - also called Hurler Syndrome (MPS IH) (OMIM: 607014) - and attenuated forms - known as Hurler-Scheie Syndrome (MPS IH-S) (OMIM: 607015) and Scheie Syndrome (MPS IS) (OMIM: 607016). The three forms are not biochemically distinguishable, and the onset of symptoms occurs at the pediatric age group. MPS I is a multisystemic disease. Its patients can show neurological, musculoskeletal, ophthalmologic, cardiac, and upper and lower airway involvement, in addition to recurrent infections. Therefore, a multidisciplinary team should monitor them.^4^

Specific therapeutic options for MPS I include enzyme replacement therapy (ERT) with laronidase (human recombinant a-L-iduronidase, Aldurazyme^®^, BioMarin/Genzyme LCC) for patients who have the attenuated form,^5^ ^,^ ^6^ ^,^ ^7^ ^,^ ^8^ and hematopoietic stem cell transplantation (HSCT) associated or not with ERT for patients with the severe form.^9^ ^,^ ^10^ ^,^ ^11^ ^,^ ^12^ ^,^ ^13^ The standard laronidase treatment regimen consists of an intravenous dose of 0.58 mg/kg (100 U/kg)/week,^14^ ^,^ ^15^ approved by regulatory agencies from the USA (Food and Drug Administration - FDA) and Europe (European Medicines Agency - EMA) in 2003 and the Brazilian Health Regulatory Agency (*Agência Nacional de Vigilância Sanitária* - ANVISA) in 2005.^16^ Efficacy studies of ERT,^14^ ^,^ ^15^ a phase IV extension study, and a safety and tolerability study in patients younger than five years^17^ used urinary GAG concentration - measured by the spectrophotometric method using methylene blue^17^ - as a biomarker.

Our service participated in a laronidase dose-optimization trial,^18^ in which one regimen comprised biweekly infusions. After 26 weeks of study, all participants were instructed to resume the standard ERT regimen at a dose of 0.58 mg/kg/week. In this dosing scheme, some parents declared difficulty in obtaining venous access and visiting the treatment center every week. Based on the positive experience of the trial participants under the regimen with biweekly infusions and the complaints of parents of patients under weekly ERT, we adopted a biweekly treatment regimen at a dose of 1.2 mg/kg every two weeks, comparing urinary GAG concentration in both the standard and biweekly schemes.

This study aimed to report the effect of a biweekly ERT regimen on the clinical response of patients and their urinary GAG excretion.

## METHOD

This is a retrospective study with data collected from medical records on urinary GAGs, adherence to treatment, and clinical information. The Research Ethics Committee (REC) of Universidade Federal de São Paulo (Unifesp) approved this study, under the number 0802/07.

All MPS I patients under ERT for over a year with no infusion associated reaction (IAR) and good adherence to the weekly regimen were eligible for the biweekly dose. Out of the eight patients who started this treatment, seven had participated in the dose-optimization trial. The laronidase dose of 1.2 mg/kg was reconstituted in 250 mL of 0.9% NaCl q.s. and administered by infusion pump at the speed recommended by the manufacturer for 4 hours every two weeks.

We collected data on the presence of cardinal signs of MPS I, such as corneal opacity, macrocephaly, hepatosplenomegaly, and joint limitation, in the standard regimen close to the change to the biweekly one and after a year of permanence in this scheme. Information about urinary GAG was gathered from patients with five consecutive infusions under the standard regimen prior to the change in dose and other five consecutive infusions after the start of the biweekly scheme. Data were collected from medical records on reports of adverse events in the 12 months prior and subsequent to the change in dose regimen. Parents and guardians are instructed to communicate the presence of a new adverse event, and not wait to inform them only during the routine medical appointment. We assessed adherence to treatment using the percentage of absences from infusions in the 12 months prior and subsequent to the change to the biweekly regimen.

In the molecular analysis, patients 1, 2, 3, and 8 were screened for the three most common mutations - W402X, Q70X, and P533R - by polymerase chain reaction (PCR) and restriction enzyme digestion.^19^ Deoxyribonucleic acid (DNA) sequencing of patient 7 was done with QIAquick Gel Extraction kit (QIAGEN, Hilder, Germany), following the manufacturer’s instructions, and sequenced using BigDye Terminator v3.1 Cycle Sequencing kit (Invitrogen, Carlsbad, CA, USA) and ABI Prism 3130xl Genetic Analyzer sequencer (Applied Biosystems, Foster City, CA, USA).

Patients under ERT were instructed to bring the first morning urine sample of the day of the infusion for the urinary GAG analysis. Urinary GAG excretion was determined using the technique described by Jong et al.,^20^ based on the colorimetric reaction of methylene blue, with the upper normal limit established for each age group listed below:

- <1 year (<35.8 mg GAG/mmol creatinine).
- 1-2 years (<16.2 mg GAG/mmol creatinine).
- 2-4 years (<14.2 mg GAG/mmol creatinine).
- 4-6 years (<9.2 mg GAG/mmol creatinine).
- 6-10 years (<9.1 mg GAG/mmol creatinine).
- 10-15 years (<6.7 mg GAG/mmol creatinine).
- 15-20 years (<4.9 mg GAG/mmol creatinine).
- 20-50 years (<3.2 mg GAG/mmol creatinine).

We measured urinary creatinine using the Biotécnica kit (Varginha, MG, Brazil).

For the statistical analysis, we used the software GraphPad Prism 5.0, (San Diego, CA, USA). The two-tailed Mann-Whitney test assessed whether the difference in urinary GAG in the two dose regimens was significant. We considered p<0.05 statistically significant.

## RESULTS

This trial included eight patients (three males and five females) with a median age of 11.4 years (6-22 years), and who were under the standard ERT regimen for 2.1±0.3 years before changing to the biweekly one. Table 1 presents the demographic data of these patients.

**Table 1:** Demographic information of patients with mucopolysaccharidosis type I.

Patient	Gender	Phenotype (H, H-S, S)	Genotype	Consanguinity	Age at onset of symptoms (years)	Age at diagnosis (years)	Age at start of ERT (years)	Age at regimen change (years)
1	M	H-S	P533R/P533R	Yes	3	14.8	16.7	18.8
2^a^	F	H	P533R/P533R	No	0.3	5.2	7.9	9.9
3^a^	F	H-S	P533R/P533R	No	2	2.6	4.6	6.5
4^b^	M	H	NP	Yes	1.3	4.9	5.9	7.9
5^b^	M	H	NP	Yes	0.7	3.1	4.1	6.1
6	F	H	NP	No	0.8	3.2	4.3	6.0
7	F	S	W402X/R383H	No	2	1.9	19.6	21.8
8	F	H-S	W402X/P533R	No	1.5	7.2	9.4	12.3

H: Hurler; H-S: Hurler-Scheie; S: Scheie; ERT: enzyme replacement therapy; M: male; F: female; NP: not performed; atwo sisters 1; btwo brothers 2.

**Figure 1:**
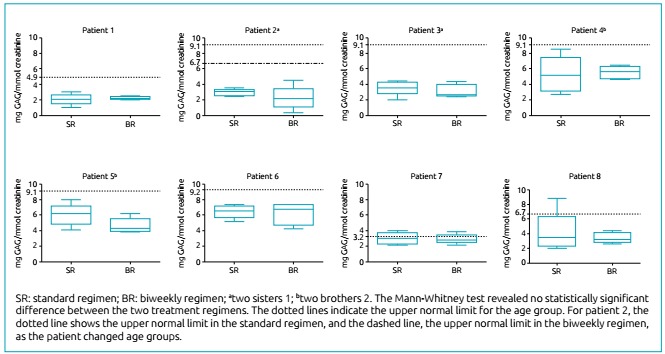
Urinary glycosaminoglycan excretion in patients with mucopolysaccharidosis type I under different laronidase dose regimens: standard regimen - 0.58 mg/kg/week; biweekly regimen - 1.2 mg/kg every two weeks.

The urinary GAG analysis showed that the values of all patients under the biweekly regimen remained stable compared to the standard one. Patient 2 changed age groups - adopted for normality reference - during the trial period, but the values from her urinary GAG analysis also remained stable under the biweekly regimen, in relation to the standard one (Figure 1 and Table 2).

**Table 2:** Concentration of urinary glycosaminoglycan excretion in patients under the standard laronidase regimen of 0.58 mg/kg/week and the alternative biweekly laronidase regimen of 1.2 mg/kg every two weeks (biweekly regimen).

Patient	1		2^a^		3^a^		4^b^		5^b^		6		7		8	
Treatment regimen	SR	BR	SR	BR	SR	BR	SR	BR	SR	BR	SR	BR	SR	BR	SR	BR
Median*	2.13	2.18	3.11	2.20	3.59	2.68	5.18	5.64	6.18	4.27	6.51	6.72	2.97	2.80	3.46	3.20
Standard deviation*	0.64	0.18	0.39	1.34	0.81	0.74	2.06	0.72	1.26	0.85	0.74	1.25	0.67	0.54	2.44	0.64
Minimum*	1.07	2.02	2.49	0.36	2.10	2.44	2.71	4.68	4.10	3.86	5.16	4.22	2.13	2.17	2.05	2.61
Maximum*	3.08	2.51	3.58	4.55	4.46	4.34	8.56	6.45	7.99	6.19	7.31	7.35	3.99	3.83	8.87	4.41
Normal for age*	4.9		9.1	6.7	9.1		9.1		9.1		9.2		3.2		6.7

SR: standard regimen; BR: biweekly regimen; *all values in mg GAG/mmol creatinine; ^a^two sisters 1; ^b^two brothers 2.

All patients had typical clinical manifestations of MPS I, such as macrocephaly, corneal opacity, and joint limitation, which remained stable after the change to the biweekly regimen, and adherence to treatment was better in this scheme (Table 3). There were no IAR during the biweekly infusion period.

**Table 3:** Clinical manifestations and adherence to the standard laronidase treatment regimen of 0.58 mg/kg/week (standard regimen) and alternative laronidase treatment regimen of 1.2 mg/kg every two weeks (biweekly regimen).

Patient	Corneal opacity		Macrocephaly		Hepatomegaly		Splenomegaly		Joint limitation		% of adherence to ERT^c^	
	SR	BR	SR	BR	SR	BR	SR	BR	SR	BR	SR	BR
1	Yes	Stable	No	Absent	No	No	No	No	Yes	Stable	84	93
2^a^	Yes	Stable	Yes	Stable	No	No	No	No	Yes	Stable	88	100
3^a^	Yes	Stable	Yes	Stable	No	No	No	No	Yes	Stable	84	93
4^b^	Yes	Stable	Yes	Stable	Yes	Stable	No	No	Yes	Stable	76	79
5^b^	Yes	Stable	Yes	Stable	No	No	No	No	Yes	Stable	72	86
6	Yes	Stable	Yes	Stable	No	No	No	No	Yes	Stable	88	100
7	Yes	Stable	No	Absent	No	No	No	No	Yes	Stable	72	93
8	Yes	Stable	No	Absent	Yes	Stable	No	No	Yes	Stable	84	100

^a^Two sisters 1; ^b^two brothers 2; ^c^evaluated 12 months before and after changing the regimen; ERT; enzyme replacement therapy.

## DISCUSSION

Weekly ERT improves many MPS I symptoms, such as hepatosplenomegaly, forced vital capacity, and gait - measured by the six-minute walk test.^8^ ^,^ ^15^ A retrospective study with 35 patients under weekly ERT, aged one to ten years (mean of 6.1 years) showed stabilization of the aortic and mitral valvular disease in 65% of subjects, corneal opacity in 78%, and visual acuity in 33%; moreover, visual acuity improved for 42% of patients.^21^

Our group has described the negative impact of ERT on family routine. Most parents or guardians complain about the time spent in the journey between home and treatment center, as well as the duration of the infusion.^22^ In another study, patients with Gaucher disease who also need ERT expressed their wish to reduce the frequency of infusions.^23^ Therefore, we changed the ERT dose regimen for MPS I patients to try to minimize the inconvenience the infusions cause in the family routine.

Urinary GAG excretion is not directly correlated with clinical manifestation or response to treatment, but phase I/II/III clinical trials used it as a biomarker,^14^ ^,^ ^15^ ^,^ ^17^ and interrupting ERT led to a fast rise in its levels and return to pre-treatment values.^24^ ^,^ ^25^ In the biweekly regimen, urinary GAG excretion did not increase, and improvement of reversible clinical parameters that patients had already achieved during the weekly scheme was not compromised. Parents and guardians of the patients declared their satisfaction in visiting the treatment center less often.

A multinational retrospective study with 20 MPS I patients compared their clinical manifestations before ERT and under laronidase treatment in standard and biweekly regimens^26^, and revealed that most participants had hepatosplenomegaly, umbilical and/or inguinal hernia, and musculoskeletal and cardiac abnormalities at the start of ERT, remaining stable or improving after initiating the standard treatment. Their condition did not get worse with the change to the biweekly regimen. Results of the urinary GAG analysis were shown in multiples of the upper normal limit. Before starting ERT, 19/20 patients had their urinary GAG data available, all above the upper normal limit, with a median 8.5 times higher than the upper normal limit (ranging from 1.7 to 148 times). After the standard treatment regimen, 13/20 patients presented urinary GAG excretion within the normal range for their age. Among the 7/20 who still showed elevated urinary GAG excretion, the median was 2.1 times the upper normal limit (ranging from 1.1 to 2.9 times). In the biweekly regimen, the urinary GAG excretion of 15/20 patients was within the normal range. Among the 5/20 who remained with elevated urinary GAG excretion, the median was 1.8 the upper normal limit (ranging from 1.2 to 3.8 times).^26^

A limitation of the present study - and others with rare diseases - was the small number of participants. Another limitation - common to all retrospective studies using data from medical records - was the lack of control of the researcher over the original data collection, which frequently leads to the loss of some information. Our intention was not to describe the most effective treatment regimen, but to verify if the biweekly regimen was not inferior to the weekly one, using the single biomarker available at that time - urinary GAG -, as up to that moment no study showed the clinical effect of a larger interval between infusions, even with twice the standard dose.

In conclusion, the biweekly laronidase regimen at 1.2 mg/kg had no negative effect on urinary GAG excretion and clinical parameters of patients who were previously under weekly ERT for more than a year remained stable. Also, laronidase is contraindicated in the initial phase of infection, leading patients with acute infection to lose some infusions since we had no time slot available in the same week to reschedule that dose. In this regard, the biweekly regimen has an advantage, as it allows the patient to reschedule the infusion for the following week, which is not possible in the standard regimen, facilitating adherence to treatment of 100%.
